# TIMP1 is a prognostic marker for the progression and metastasis of colon cancer through FAK-PI3K/AKT and MAPK pathway

**DOI:** 10.1186/s13046-016-0427-7

**Published:** 2016-09-20

**Authors:** Guohe Song, Shifeng Xu, Hong Zhang, Yupeng Wang, Chao Xiao, Tao Jiang, Leilei Wu, Tao Zhang, Xing Sun, Lin Zhong, Chongzhi Zhou, Zhaowen Wang, Zhihai Peng, Jian Chen, Xiaoliang Wang

**Affiliations:** 1 Department of General Surgery, Shanghai General Hospital, School of Medicine, Shanghai Jiao Tong University, Shanghai, 200080 People’s Republic of China; 2 Department of General Surgery, Shandong Provincial Hospital Affiliated to Shandong University, Jinan, Shandong 250021 People’s Republic of China; 3 School of Medicine, Örebro University, Örebro, SE 70182 Sweden; 4 School of Life Sciences and Biotechnology, Shanghai Jiao Tong University, Shanghai, 200031 People’s Republic of China

**Keywords:** TIMP1, Colon cancer, Prognosis, Tumorigenesis

## Abstract

**Background:**

Tissue inhibitor matrix metalloproteinase 1 (TIMP1) plays a vital role in carcinogenesis, yet its precise functional roles and regulation remain unclear. In this study, we aim to investigate its biological function and clinical significance in human colon cancer.

**Methods:**

We analyzed the expression of TIMP1 in both public database (Oncomine and TCGA) and 94 cases of primary colon cancer and matched normal colon tissue specimens. The underlying mechanisms of altered TIMP1 expression on cell tumorigenesis, proliferation, and metastasis were explored in vitro and in vivo.

**Results:**

TIMP1 was overexpressed in colon tumorous tissues and lymph node metastasis specimens than in normal tissues. The aberrant expression of TIMP1 was significantly associated with the regional lymph node metastasis (*p* = 0.033), distant metastasis (*p* = 0.039), vascular invasion (*p* = 0.024) and the American Joint Committee on Cancer (AJCC) stage (*p* = 0.026). Cox proportional hazards model showed that TIMP1 was an independent prognostic indicator of disease-free survival (HR = 2.603, 95 % CI: 1.115–6.077, *p* = 0.027) and overall survival (HR = 2.907, 95 % CI: 1.254–6.737, *p* = 0.013) for patients with colon cancer. Consistent with this, our findings highlight that suppression of TIMP1 expression decreased proliferation, and metastasis but increased apoptosis by inducing TIMP1 specific regulated FAK-PI3K/AKT and MAPK pathway.

**Conclusion:**

TIMP1 might play an important role in promoting tumorigenesis and metastasis of human colon cancer and function as a potential prognostic indicator for colon cancer.

**Electronic supplementary material:**

The online version of this article (doi:10.1186/s13046-016-0427-7) contains supplementary material, which is available to authorized users.

## Background

Colon cancer represents one of the most common malignancies worldwide and a common cause of morbidity and mortality [1]. Despite increased treatment advances in the past 20 years, early diagnosis can still improve the prognosis of this disease [2]. Molecular studies have revealed a large number of genetic alterations that occur during colon carcinogenesis, however, precise genetic changes responsible for the occurrence and progression of colon cancer is still poorly understood [3, 4]. Therefore, identification of molecular markers remains crucial for designing novel and efficient treatment strategy.

Tissue inhibitor matrix metalloproteinase 1 (TIMP1), located on chromosome Xp11.3-p11.23, belongs to the Tissue Inhibitor of Metalloproteinases family which included four identified members (TIMP1, TIMP2, TIMP3, and TIMP4). TIMP1 encodes a 931 base-pair mRNA and a 207 amino acid protein. Studies have shown that this protein may inhibit the proteolytic activity of matrix metalloproteinases (MMPs) by forming noncovalent 1:1 stoichiometric complexes and regulate the balance of matrix remodeling during degradation of extracellular matrix [5]. In addition to its inhibitory effect on most of the known MMPs, which are thought to be crucial for the tumor invasion and development of metastatic disease [6], TIMPs also play an important role in the regulation of cell proliferation and anti-apoptotic function [7–9]. Studies in vitro have demonstrated that overexpression of TIMP1 can lead to a substantially increase of genes involved in proliferation, apoptosis and signal transduction [5]. In addition, TIMP1 was found to decrease tumor cell sensitivity to multiple anticancer drugs by activation of downstream pathways and exhibited anti-apoptotic activity. Specifically, the TIMP1 could degrade cyclinB1 and activate the NF-kβ signaling pathway to protect breast cancer cells against chemotherapy-induced cell death [10]. In particular, it has also been demonstrated that TIMP1 might bind to the CD63/integrin β1 complex causing anti-apoptotic effects [11].

Recently, clinical studies have shown that the aberrant expression of TIMP1 is associated with an unfavorable prognosis in a series of tumors, such as gastric cancer [12], papillary thyroid carcinoma [13], cutaneous melanoma [14] and breast cancer [8]. The expression level of TIMP1 often correlates with the TNM stage, disease-free survival, the rate of tumor recurrence and even the extent of liver metastasis. Furthermore, TIMP1 is a secretory protein that could be detected in blood and body fluid by enzyme linked immunosorbent assay (ELISA). The expression level of TIMP1 was significantly increased in the blood of patients with gastric cancer [12] and familial pancreatic cancer [15], which could make it as a potential serum marker. However, the precise function and underlying mechanisms of TIMP1 remain to be elucidated in colon cancer.

In the present study, we investigate the expression of TIMP1 at the mRNA and protein level in human colon cancer and clarify the correlation between the TIMP1 expression and clinicopathological parameters. We also sought to determine the role of TIMP1 in colon cancer cell biological function and the underlying molecular mechanisms.

## Methods

### Patient information and clinical specimens

A total of 94 patients who had surgery for colon cancer between January 2007 and December 2009 at the Shanghai General Hospital were selected for this study. This study was approved by the Institutional Research Ethics Committee of Shanghai General Hospital. Patients received neither chemotherapy nor radiotherapy before surgery. There were 47 male and 47 female with a median age of 67 years (range, 36–92 years) at the time of operation. The tumor stage classification was determined by at least two pathologists who were blinded to the data according to the American Joint Committee on Cancer (AJCC). Disease-free survival (DFS) and overall survival (OS) rates were defined as the interval from initial surgery to clinically or radiologically proven recurrence/metastasis and death, respectively. Cancer tissues and metastatic lymph nodes obtained from patients were immediately fixed in formalin and embedded in paraffin for immunohistochemical (IHC) studies after dehydration. Fresh samples were dissected manually and were immediately stored in liquid nitrogen until western blot analysis. Written and informed consent was obtained from all patients before enrollment in the study.

### The Oncomine,Cancer Genome Atlas (TCGA) and Protein-Protein Interaction (PPI) network

Oncomine (www.oncomine.org) was used to acquire TIMP1 RNA expression in colon carcinoma and normal mucosae. TCGA RNA-Seq (level 3) and corresponding clinical data were downloaded from TCGA website (https://tcga-data.nci.nih.gov/tcga/) following approval of this project by the consortium. RNA-Seq analysis used data from 45 stage I, 108 stage II, 80 stage III and 38 stage IV colon cancers and 40 adjacent normal tissues. To further analyze the function of the significant biomarkers, the Retrieval of Interacting Genes/Proteins (STRING database v10.0) resource was utilized for PPI network analysis and prediction of protein-protein interactions. Proteins were linked based on the following six criteria: neighborhood, gene fusion, co-occurrence, co-expression, experimental evidence and existing databases. A p value of less than 0.05 was considered as the cut-off criterion. TIMP1’s PPI network containing the top 20 confident proteins were built in Additional file 1: Figure S1.

### Gene Set Enrichment Analysis (GSEA)

GSEA is a method of analyzing and interpreting microarray and such data using biological knowledge. The data firstly generated an ordered list of all genes according to their correlation with TIMP1 expression, and then a predefined gene set receives an enrichment score (ES), which is a measure of statistical evidence rejecting the null hypothesis that its members are randomly distributed in the ordered list. GSEA was performed using The Cancer Genome Atlas (TCGA) colorectal cancer (CRC) dataset by GSEA version 2.2 from the Broad Institute at MIT.

### Western blot assays

Total protein was extracted from colon tumors and their adjacent normal tissues of 4 patients using RIPA lysis buffer with inhibitor phenylmethanesulfonyl fluoride (PMSF) and then the concentration was measured with BCA protein assay kit (Beyotime Biotechnology, Jiangsu, China). Equal amounts of protein (30 μg) were subjected to 10 % sodium dodecyl sulfate-polyacrylamide gelelectrophoresis and then transferred onto PVDF membranes. The membranes were blocked in 5 % non-fat milk with 0.1 % Tween for 1 h at room temperature followed by incubating at 4 ^∘^C overnight with primary rabbit polyclonal antibody against human protein TIMP1 (1:1000 dilution, Abcam, USA) and β-actin (1:1000 dilution, Abcam, USA). After washing with TBST buffer, the membrane was incubated with secondary antibodies conjugated to horseradish peroxidase. The anti-β-actin antibody was used as loading controls. The proteins were detected by ECL chemiluminescence kit (Pierce Biotechnology, Rockford, IL, USA) according to the manufacturer’s instructions.

### Immunohistochemical analysis

Sections (4-μm thick) were blocked with 3 % H_2_O_2_ after dewaxing and rehydrating in a graded series of ethanol. Then, pressure cooker mediated antigen retrieval process was performed in 0.01 M sodium citrate solution (pH 6.0) and the expression of TIMP1 was tested using standard immunohistochemical methods [16]. The corresponding primary antibody was used as follows,TIMP1 (1:100 dilution, Abcam, USA). Immunoreactivity was evaluated independently by two pathologists who were blinded to patient information. The evaluation was based on the staining intensity and the area of staining. The staining intensity was graded as follows: 0, no staining; 1, mild staining; 2, moderate staining; and 3, intense staining. The staining area was scored as follows: 0, no staining of cells; 1, 1–25 %; 2, 26–50 %; 3, 51–75 %; and 4, 76–100 %. The sum of staining score (intensity and extension) index was used as the final staining score, graded as follows: 0–1, negative expression; 2–4, weakly positive expression; and 5–6, strongly positive expression. Weakly positive and strongly positive were considered as positive.

### Plasmids construction, transfections and lentiviral transduction

The control shRNA lentivirus, and TIMP1 shRNA lentivirus were all constructed by Shanghai Genechem Medical Biotechnology Company.

siRNA-Control Target: AATTCTCCGAACGTGTCACGT;

Sense: UUCUCCGAACGUGUCACGUdTdT;

Antisense: ACGUGACACGUUCGGAGAAdTdT;

siRNA-TIMP-1 Target: ATCAACCAGACCACCTTATA;

Sense: UCAACCAGACCACCUUAUAdTdT;

Antisense: UAUAAGGUGGUCUGGUUGAdTdT;

For lentivirus transfections, colon cancer cells were transfected with 5 μg of lenti-TIMP1-virus or lenti-control virus. Methods used for lentivirus production and infection were performed as described previously [16].

### RNA extraction, reverse transcription PCR and quantitative real-time PCR (qPCR)

Total RNA was prepared from cell cultures using TRIzol reagent (TaKaRa, Japan) according to the manufacturer’s instructions. First strand cDNA was synthesized from 2 μg of total RNA using RevertAid™First Strand cDNA Synthesis Kit (Fermentas, USA). The quantitative real-time PCR primers were provided by Sheng Gong Company. The sequences of forward and reverse primers are used as follows:

TIMP1-forward: 5′-CGCAGCGAGGAGGTTTCTCAT-3’;

TIMP1-reverse: 5′-GGCAGTGATGTGCAAATTTCC-3’.

β-actin-forward: 5′-TCCTTAATGTCACGCACGATTT-3’;

β-actin-reverse: 5’-GAGCGCGGCTACAGCTT-3’;

The reaction was performed for 40 cycles of 95 °C for 2 min, and then 95 °C for 10 s, 60 °C for 30 s, and 72 °C for 30 s, with a final extension at 72 °C for 30 s. Relative quantities (Δ cycle threshold (Ct) value) were obtained by normalizing to β-actin. Each reaction was performed in triplicate.

### Cell proliferation and plate colony formation assays

Cells were seeded into 96-well plates (2000 cells per well), cultured for 24 h, and then measured using 10 μl Cell Counting Kit-8 (CCK-8; Dojindo Laboratories, Japan) in 100 μl medium per well for 1 h at 37 °C, and then absorbance was measured at 450 nm. For plate colony formation assays, 800 cells were seeded in six-well plates and cultured at 37 °C under a humidified atmosphere containing 5 % CO_2_ for 2 weeks. The cells were then fixed with methyl alcohol for 30 min and stained with Giemsa solution for 10 min. Colonies were then counted and photographed. All experiments were performed in triplicate.

### Cell scratch-wound assay

Cells were grown in 6-well plates until confluence. A wound was generated by scraping with a 10 ul tip. After 12 h, the cells in the wounded monolayer were photographed and cell migration was assessed by measuring gap sizes at multiple fields.

### Tumor cell invasion assays

Boyden chambers with filter inserts (pore size, 8-μm) coated with Matrigel in 24-well dishes (BD Biosciences, USA) was performed to detect tumor cell invasion assays. Colon cancer cells with different treatment were then harvested, and 1 × 10^5^ cells were seeded in serum-free medium into the upper chamber, whereas medium supplemented with 20 % fetal bovine serum was applied to the lower chamber as a chemoattractant to induce invasion. The migrated cells on the lower side of the filters were defined as invasive cells and counted at × 200 magnification in 10 different fields of each filter.

### Confocal laser scanning microscopy

Cells were seeded into 35 mm glass bottom dishes with 10 mm microwell overnight. After permeabilized with 0.3 % Triton X-100 diluted in PBS for 15 min and then blocked with 5 % skimmed milk for 1 h, cells were incubated with primary antibody at 4 °C overnight. After washed with PBS three times for 5 min each time, cells were subjected to fluorescent secondary antibody at room temperature for 1 h. Before confocal microscopy analysis, cells were counterstained with DAPI.

### Flow cytometric analysis

The apoptotic status was analyzed by using an Annexin V-APC/PI Apoptosis Kit (eBioscience, CA, USA) in accordance with the manufacturer’s instructions. Briefly, cells (1 × 10^6^cells/mL) were collected by EDTA treatment and incubated with a mixture of annexin V-FITC and PI for 15 min at room temperature. The number of apoptotic cells was analyzed by flow cytometry (BD Accuri C6, USA).

### Animal experiments

Male BALB/c athymic nude mice (4–6 weeks old) purchased from the Institute of Zoology, Chinese Academy of Sciences of Shanghai. All mice were injected subcutaneously into the right side of back with 1.0 × 10^7^ cells to establish the CRC xenograft model. One week after first injection, the two groups were sacrificed. Tumor diameters were measured at regular intervals with digital calipers, and tumor volume was calculated by the formula: tumor volume (mm^3^) = shorter diameter^2^ × longer diameter/2. To further investigate the metastasis effect of TIMP1 in vivo, we used a CRC metastasis model in male BALB/c-nu/nu mice. A total of 1 × 10^6^ cells were infected with lenti-LUC virus and injected into the tail veins of nude mice. In weekly intervals, anesthetized mice were injected i.p. with D-luciferin (150 mg/kg) and imaged 10 min after injection using the IVIS Illumina System (Caliper Life Sciences).

### Statistical analysis

Gene expression analysis was performed using R (http://www.r-project.org). Data from two experimental groups were compared with the *t*-test, chi-square test or Fisher’s exact test, as appropriate. Survival curves were drawn using the Kaplan-Meier method. The differences of the Kaplan-Meier survival curves were tested for statistical significance with the log rank test, and the 95 % confidence intervals were calculated. Multivariate analysis was done using the COX proportional hazard model and a forward stepwise method was used to bring variables into the model. Further statistical analyses were done using the SPSS 21.0 software (SPSS Inc.). A significant difference was declared if the p value from a two-tailed test was less than 0.05.

## Results

### TIMP1 expression is significantly upregulated in human colon cancer

**Fig. 1:**
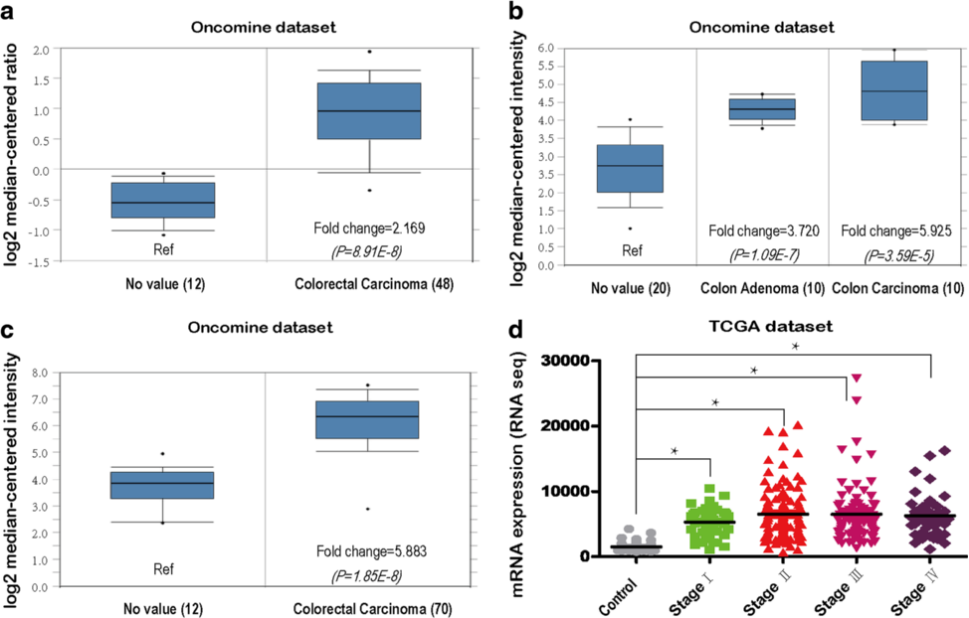
Analysis of TIMP1 RNA expression on the basis of the Oncomine and Cancer Genome Atlas (TCGA) database. **a**-**c** Exhibition of TIMP1 expression of normal specimens and colorectal carcinoma from colorectal statistics in Oncomine dataset (**a** Graudens E, 2006; **b** Skrzypczak M, 2010; **c**, Hong Y, 2010). The expression fold changes were 2.169, 5.929 and 5.883 times, respectively, all *p* < 0.01. **d** TCGA dataset showed that the TIMP1 level was increased in stages I, II, III, and IV compared with that in adjacent normal mucosa

**Fig. 2:**
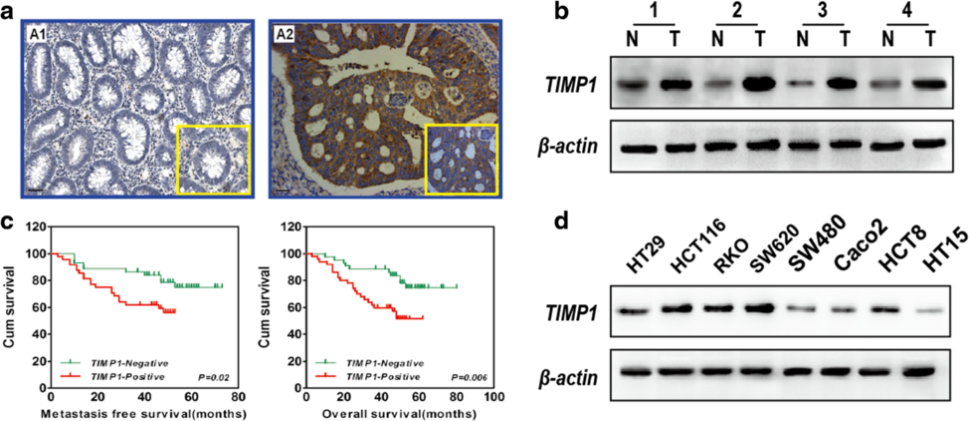
TIMP1 expression and pathologic features in colorectal cancer specimens. **a** Immunohistochemical staining of TIMP1. TIMP1 was localized within the cytoplasm and overexpression in the tumor cells of colorectal cancer tissue. **b** Western blot analysis was performed to assess TIMP1 protein levels in 4 representative cases of colorectal cancer specimens. **c** The disease-free survival (DFS) and overall survival (OS) rates were estimated by the Kaplan–Meier method. Both the DFS rate and OS rate of patients with TIMP1 positive primary tumor were significantly lower than that of patients with TIMP1 negative primary tumor. **d** TIMP1 protein levels in colorectal cancer cell lines (scale bar = 40 μm)

We first browse Oncomine and found that the expression level of TIMP1 was significantly high in tumor tissues compared with the related normal mucosae [17–19] (Fig. 1a-c). Furthermore, we analyzed data from The Cancer Genome Atlas (TCGA) according to AJCC stage. The expression data of TIMP1 genes from these patients, obtained in RNA-Seq experiments showed that the expression level of TIMP1 in colon patients was augmented in stages I, II, III, and IV compared with that in adjacent normal mucosa (Fig. 1d). Western blot revealed that the protein levels of TIMP1 were differentially upregulated in all 4 colon cancer samples compared to the matched adjacent non-tumor tissues (Fig. 2b).

### Correlation between TIMP1 expression and the clinical features of colon cancer

To determine whether TIMP1 is clinically correlated with colon cancer progression, the expression of TIMP1 was determined in a tissue microarray containing 94 cases of primary colon tumors paired with their normal colon mucosa tissue and 36 lymph node metastases (LNM) specimens by immunohistochemistry. Among the 94 samples on the paired TMA, 57 (60.6 %) showed negative staining in normal mucosa. In contrast, TIMP1 was prominently expressed in primary tumor specimens, with strong staining in 26 (27.7 %) samples, weak staining in 24 (25.5 %) samples, and negative staining in 44 (46.8 %) samples (Table 1). Of the 36 samples of available lymph node metastasis specimens, 29 samples (80.6 %) showed TIMP1 overexpression (Table 1). Positive staining of TIMP1 protein expression was observed mainly in the cytoplasm of the cancer cells (Fig. 2a).

**Table 1:** Expression of TIMP1 protein in normal mucosa, cancerous tissue, and LNM tissues

TIMP1 expression					
Tissue sample	n	Negative	Weak	Positive	*P*-value
Normal mucosa	94	57 (60.6 %)	22 (23.4 %)	15 (16.0 %)	<0.01*
Cancerous tissue	94	44 (46.8 %)	24 (25.5 %)	26 (27.7 %)	
LNM tissue	36	7 (19.4 %)	11 (30.6 %)	18 (50.0 %)	

*LNM* lymph node metastasis

*p-value is based on chi-square test

Summarization of the relationship between expression levels of TIMP1 and clinical parameters is shown in Table 2. Our study revealed that upregulated TIMP1 level was significantly correlated with the regional lymph node metastasis (*p* = 0.033), distant metastasis (*p* = 0.039), vascular invasion (*p* = 0.024) and the AJCC stage (*p* = 0.026), while no association was found between TIMP1 expression and age, gender, tumor location, T-classification or differentiated degree.

**Table 2:** Correlation between TIMP1 expression and clinicopathological characteristics

	Total (*n* = 94)	TIMP1 protein expression		*P*-value
		Negative (*n* = 44)	Positive (*n* = 50)	
Age				
< 65	43 (45.7 %)	21 (47.7 %)	22 (44.0 %)	0.717
≥ 65	51 (54.3 %)	23 (52.3 %)	28 (56.0 %)	
Gender				
Male	47 (50.0 %)	23 (52.3 %)	24 (48.0 %)	0.679
Female	47 (50.0 %)	21 (47.7 %)	26 (52.0 %)	
Tumor location				
Right	25 (26.6 %)	12 (27.3 %)	13 (26.0 %)	0.423
Transverse	9 (9.6 %)	5 (11.4 %)	4 (8.0 %)	
Left	10 (10.6 %)	6 (13.6 %)	4 (8.0 %)	
Others	50 (53.2 %)	21 (47.7 %)	29 (58.0 %)	
T stage				
T1 + T2	23 (24.5 %)	10 (22.7 %)	13 (26.0 %)	0.713
T3 + T4	71 (75.5 %)	34 (77.3 %)	37 (74.0 %)	
N stage				
N0	51 (54.3 %)	29 (65.9 %)	22 (44.0 %)	0.033*
N1 + N2	43 (45.7 %)	15 (34.1 %)	28 (56.0 %)	
M stage				
M0	80 (85.1 %)	41 (93.2 %)	39 (78.0 %)	0.039*
M1	14 (14.9 %)	3 (6.8 %)	11 (22.0 %)	
Vascular invasion				
No	85 (90.4 %)	43 (97.7 %)	42 (84.0 %)	0.024*
Yes	9 (9.6 %)	1 (2.3 %)	8 (16.0 %)	
Differentiation				
Well	27 (28.8 %)	9 (20.4 %)	18 (36.0 %)	0.186
Moderate	49 (52.1 %)	27 (61.4 %)	22 (44.0 %)	
Poor	18 (19.1 %)	8 (18.2 %)	10 (20.0 %)	
AJCC stage				
I + II	42 (44.7 %)	25 (56.8 %)	17 (34.0 %)	0.026*
III + IV	52 (55.3 %)	19 (43.2 %)	33 (66.0 %)	

*p < 0.05 indicates a significant association between the variables

### Overexpression of TIMP1 predicts poor clinical outcome in human colon cancer

Kaplan-Meier survival analysis with the log-rank test for disease-free survival (DFS) and overall survival (OS) were undertaken to evaluate the relationship between TIMP1 expression and patient prognosis (Fig. 2c). The analysis revealed that patients with high levels of TIMP1 possessed shorter disease-free survival (DFS) time and overall survival (OS) time than patients with low levels of TIMP1 (*p* = 0.02 and *p* = 0.006, respectively).

Multivariate analysis was performed using the Cox proportional hazards model for AJCC stage, TNM stage and vascular invasion, and the expression of TIMP1 was found to be an independent prognostic indicator to predict patient outcomes (Table 3). A significant association was found between the high TIMP1 expression level and lower 5-year DFS (HR =2.603, 95 % CI: 1.115–6.077, *p* = 0.027) and OS survival (HR = 2.907, 95 % CI: 1.254–6.737, *p* = 0.013). This suggests that TIMP1 is of clinical significance in the diagnosis and prognosis of patients with colon carcinomas.

**Table 3:** Multivariate Cox proportional hazards analysis for disease-free survival (DFS) and overall survival (OS)

Variable	Multivariate analysis (DFS)		Multivariate analysis (OS)	
	*P*-value	HR (95%CI)	*P*-value	HR (95%CI)
AJCC stage (I/II vs. III/IV)	0.037*	2.621 (1.011–6.668)	0.029*	2.915 (1.113–7.634)
N stage (N0 vs. N1 + N2)	0.043*	2.354 (1.027–5.395)	0.033*	2.785 (1.086–7.141)
M stage (M0 vs. M1)	0.049*	3.649 (1.008–13.214)	0.035*	2.465 (1.067–5.696)
Vascular invasion (Yes vs. No)	0.038*	2.488 (1.051–5.891)	0.024*	2.945 (1.151–7.535)
TIMP1 expression (Negative vs. Positive)	0.027*	2.603 (1.115–6.077)	0.013*	2.907 (1.254–6.737)

*AJCC* American Joint Committee on Cancer, *HR* hazard ratio, *CI* confidence interval

*p < 0.05 indicates a significant association between the variables

### Knockdown of TIMP1 inhibits the proliferation of colon cancer cell

**Fig. 3:**
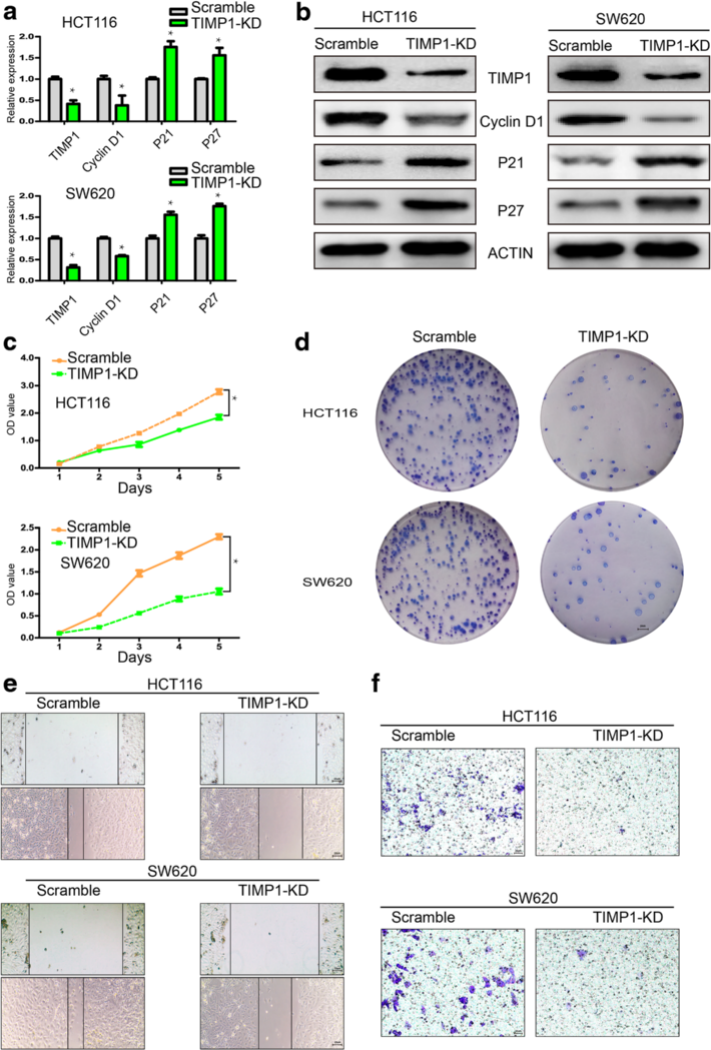
Functional roles of TIMP1 *in vitro*. **a** QPCR showed that TIMP1 was knocked down in HCT116 and SW620 cells; cyclinD1 was decreased while p21 and p27 was upregulated in TIMP1-KD cells. **b** western blot confirmed that TIMP1, CyclinD1 was decreased while P21 and P27 was upregulated in TIMP1-KD cells. **c** TIMP1-KD inhibited CRC cell growth *in vitro*. **d** TIMP1-KD inhibited CRC cell colony formation *in vitro*. **e** Cell would healling ability was impaired in TIMP1-KD cells (200×). **f** Chamber invision ability was damaged in TIMP1-KD cells (200×) (scale bar = 20 μm)

To further investigate the effects of TIMP1, shRNA was used to knockdown TIMP1 in HCT116 and SW620 colon cell lines in which TIMP1 expression level was higher than other cell lines (Fig. 2d). The efficacy of TIMP1 knockdown was confirmed by real-time PCR and western blot analysis (Fig. 3a, b). To evaluate the effects of TIMP1 knockdown (TIMP1-KD) on colon cell proliferation, the expression of proliferation-related genes (cyclinD1, p21 and p27) was detected by real-time PCR and western blot analysis (Fig. 3a, b). The expression of cyclinD1 was downregulated, while P21 and P27 was upregulated in TIMP1-KD cells. Next, we performed CCK-8 and plate colony formation assays to assess the role of TIMP1 in colon cell growth which showed that TIMP1-KD was associated with significantly decreased cell proliferation and impaired colony formation ability compared with that of scramble cells (Fig. 3c, d, *p* < 0.01). These results indicated that TIMP1 plays an important role in colon cell proliferation.

### Effects of suppression of TIMP1 in migration and invasion

**Fig. 4:**
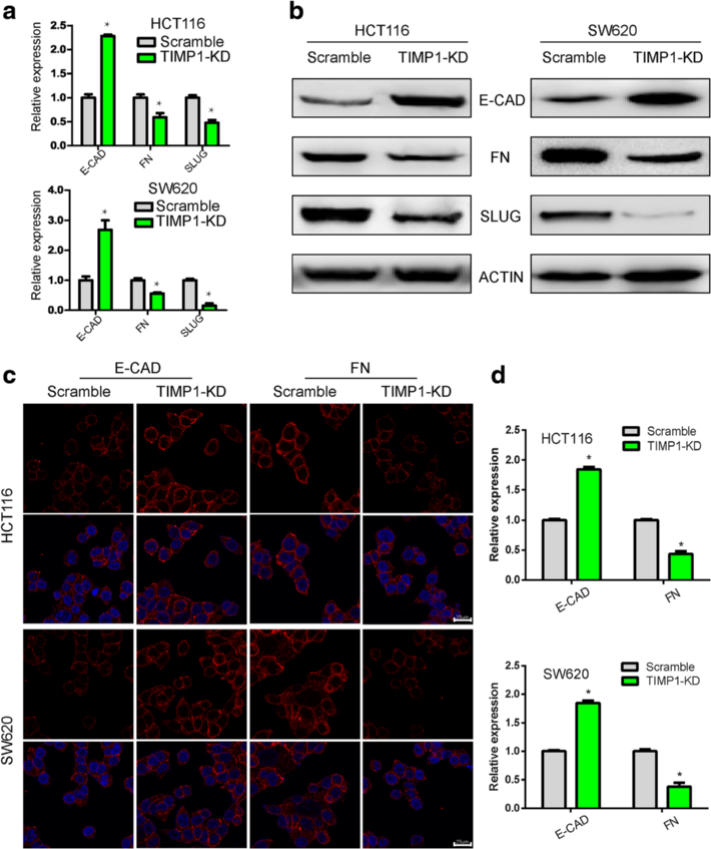
TIMP1 promotes CRC metastasis through EMT. **a** QPCR showed that E-cadherin was upregulated while SLUG and Fibronectin was downregulated in TIMP1-KD cells. **b** Western blot confirmed that E-cadherin was upregulated while SLUG and Fibronectin was downregulated in TIMP1-KD cells. **c** Immunofluorescence showed that E-cadherin was upregulated while Fibronectin was downregulated in TIMP1-KD cells **d** The relative expression of E-cadherin and Fibronectin in Scramble and TIMP1-KD cells (scale bar = 25 μm)

In wound healing assays, less wound closure was observed in TIMP1-KD colon cells when compared with scramble group (Fig. 3e). These data further suggest that TIMP1 plays an important role in cell migration. In transwell cell invasion assays, representative data showed that TIMP1-KD dramatically decreased the invasive ability of colon cells (Fig. 3f), indicating that TIMP1 may have a significant effect on cell migration and invasion. Furthermore, knockdown of TIMP1 significantly decreased SLUG, one important EMT transfactor, which lead to ascending of E-cadherin, the epithelial marker, and decline of Fibronectin, the mesenchymal marker (Fig. 4a, b). Immunofluorescence staining also confirmed the knock down effect of TIMP1 on the downregulation of Fibronectin and upregulation of E-cadherin (Fig. 4c), suggesting that TIMP1 may mediate epithelial–mesenchymal transition (EMT) initiation and colon progression.

### TIMP1 plays an anti-apoptosis role in colon cells

**Fig. 5:**
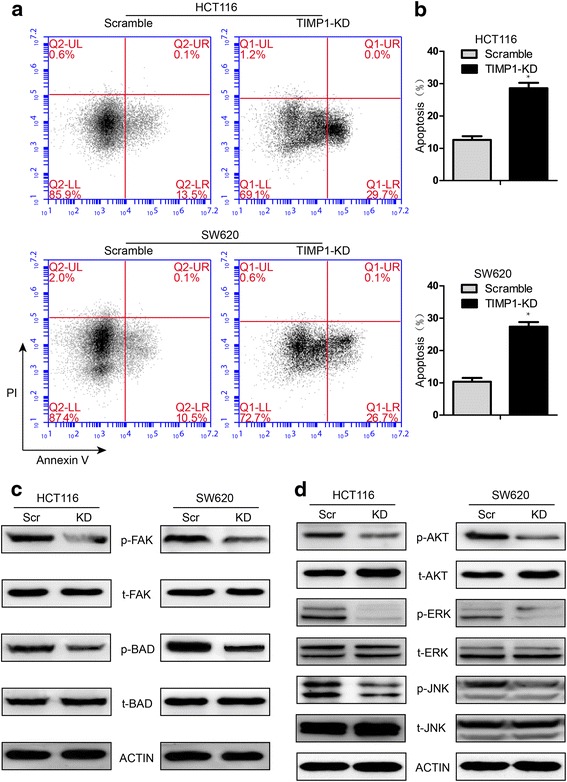
Effects of TIMP1 on apoptosis and its related pathway. **a-b** TIMP1-KD significantly increased the apoptosis rate of CRC cells; **c** Levels of pFAK/FAK, pBAD/BAD were detected in Scramble and TIMP1-KD cells. **d** Western blot of pAKT/AKT, pERK/ERK, pJNK/JNK in Scramble and TIMP1-KD cells

To test whether expression of TIMP1 affects apoptosis in colon cells, we performed an apoptosis assay by flow cytometric analysis. TIMP1-KD resulted in a significant increase in the percentage of apoptotic cells, compared with the scramble group, in both HCT116 and SW620 (Fig. 5a, b) cells. Moreover, TIMP1-KD dramatically reduced the phosphoration of BCL2-Associated Agonist Of Cell Death (BAD) (Fig. 5c), which play an important role in apoptosis [20]. When BAD is phosphorylated, it forms the BAD-(14-3-3) protein heterodimer. This leaves Bcl-2 free to inhibit Bax-triggered apoptosis [21, 22]. BAD phosphorylation is thus anti-apoptotic, and BAD dephosphorylation is pro-apoptotic [22]. These results suggest that TIMP1 could increase anti-apoptosis of colon cancer in BAD mediated phosphoration pathway.

### Mechanisms of TIMP1 in colon cancer progression

To gain further insight into the biologic pathways involved in colon cancer pathogenesis, GSEA analysis was performed in TCGA datasets. Among all the predefined ‘oncogenic signature’ gene sets, the EMT pathway was identified with the strongest association with TIMP1 expression (Additional file 2: Figure S2). The other top-scoring hallmarks included angiogenesis, apoptosis, KRAS, hypoxia and so on. Several of these hallmarks are related to PI3K and MAPK signaling pathways, activation of which are frequently aberrantly activated in human cancers and contributes to enhance cell proliferation and survival. As shown in Fig. 5c and d, transfection with TIMP1-KD significantly decreased FAK, AKT, ERK1/2 and JNK phosphorylation in both HCT116 and SW620 cells; however, no detectable changes in the total levels of FAK, AKT, ERK1/2 and JNK were observed (Fig. 5d). Thus, the data suggested that the FAK-PI3K/AKT and MAPK pathway might participate in TIMP1-induced cell proliferation, metastasis and anti-apoptosis in colon cells. To illustrate its co-worked genes, PPIs network was built from the STRING resource (Additional file 1: Figure S1). Result shows that TIMP1, was highly correlated with several stars molecules, such as MMPs, EGFR, JUN, TGF-β and SMADs, etc. This hints that TIMP1 would be a crucial marker which is valuable for further research.

### The functional impact of TIMP1 knockdown in colon cell invasion in vivo

**Fig. 6:**
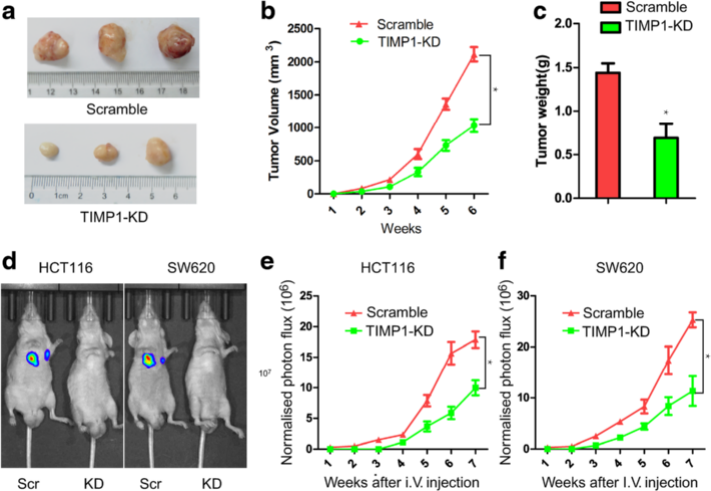
TIMP1 promotes CRC progression and metastasis in nude mice. **a** Representative Data showed that TIMP1-KD significantly inhabited tomor growth in nude mice xenograft. **b**-**c** Tumor volume and tumor weight was decreased in TIMP1-KD cells’ mice model. **d** Representative formation of lung metastases by tail-vein injection of scramble and HCT116/SW620–Luc2 cells in immunocompromised mice. Representative images of luciferase signals (*Left panel*). Normalized photon flux (*Right panel*). **e**-**f** HCT116/SW620-luc2 cells were injected into the tail vein of NOD/SCID mice, and followed by noninvasive bioluminescence imaging for 7 weeks

We further tested whether knockdown of TIMP1 could suppress the tumorigenicity of colon cells in vivo using a xenograft model in the nude mice. As shown in Fig. 6a, the tumors injected with TIMP1-KD were significantly smaller than the tumors treated with scramble cells. Tumor growth and tumor weight was significantly inhibited in TIMP1-KD injected tumors (Fig. 6b, c, *p* < 0.01), indicating that TIMP1 exert an oncogenic role during colon progression. We then explored the functional impact of TIMP1 on metastasis in vivo using a nude mouse metastatic tumor models. Different stable cells were transplanted into BALB/c-nu/nu mice via tail vein injection. We found that less subcutaneous metastasis were detected after injection of TIMP1-KD cells when compared with the injection of scramble cells (Fig. 6d-f). These data suggest that TIMP1 expression plays a critical role in colon cell motility and metastasis.

## Discussion

TIMP1, which is a kind of soluble protein release by endometrial cells, fibroblasts and cancer cells, has been demonstrated to be associated with poor prognosis for a variety of cancers [12, 23, 24]. To find the involvement of TIMP1 in colon cancer, public data, such as Oncomine and TCGA, was used to evaluate the expression of this gene in colon patients, and data from 94 patients with primary colon cancer also present with elevated levels of TIMP1 which has the potential to predict the survival rate of patients. In this study, analysis of TIMP1 expression level and patients’ clinical outcomes suggested that those with high TIMP1 expression had poorer survival than those with low TIMP1 expression. Remarkably, TIMP1 is a secretory protein that could be detected in blood and body fluid, thus making it a good potential tumor marker. However, the significance of TIMP1 and its mechanism of action in the progression of colon cancer have been rarely reported.

Despite some researches have proved that TIMP-1 has a major role as inhibitor of MMPs [25], its role in tumor invasion and metastasis appears to be more complex. It has been believed that TIMP-1 has a dual function, one of which is based on MMPs dependent anti-proteolytic activity with the other MMP-independent cell growth activity [26]. In the present study, our experiments showed that depletion of TIMP1 could suppress colon cancer cell proliferation, migration and invasion in vitro, and suppress the tumorigenicity and metastasis of colon cells in vivo. In addition, TIMP1 could also increase anti-apoptosis of colon cancer in BAD mediated phosphoration pathway. These results suggest that TIMP1 plays an important role in proliferation, invasion and metastasis of colon cancer and that TIMP1 may be a crucial therapeutic target.

To find the involved mechanism of TIMP1 in colon cancer, public data, such as TCGA, and high throughput analysis, such as STRING and GSEA was used to locate the pathway and correlated molecules which are related to TIMP1. These bioinformatics significantly pointed the function of TIMP1 to EMT, apoptosis and several other crucial pathways. To validate these predictions, biological experiment was conducted. We showed that TIMP1 overexpression is associated with constitutive activation of FAK, a signaling molecule known to be critical to the cell survival pathway [27]. FAK was shown to be the upstream regulator of the PI3K-AKT pathway [28, 29]. Phosphorylation of AKT could upregulate cyclin D1 and downregulate the cyclin D kinase (CDK) inhibitors p21 and p27, which promotes cell cycle progression. Besides, AKT phosphorylates BAD, which no longer interacts with Bcl-2 (anti-apoptotic members of the bcl-2 family), resulting in Bcl-2 activation. Similarly, MAPK family can also phosphorylate BAD protein, resulting in release of anti-apoptotic Bcl-2 in the cells (Additional file 3: Figure S3). Although TIMP1 could inhibit MMP facilitated breakdown of the ECM and was thought to be metastasis inhibitor [30], we found that TIMP1 could promote colon cancer invasion and metastasis by development of EMT transcription factors such as SLUG, leading to downregulation of epithelial marker E-cadherin and upregulation of mesenchymal marker fibronectins. In conclusion, by analyzing data from public and our own, we clearly illustrated that TIMP1 actually plays a great role in cancer progression, which is independent of MMP, and the FAK - PI3K/AKT and MAPK pathway might play an important role in TIMP1-induced cell proliferation, metastasis and anti-apoptosis. Besides, the paradoxical report of TIMP1 as an inhibitor of MMPs and poor prognosis marker of colon cancer as well, could be explained by its MMP independent function, which suggests the elevation of TIMP1 in colon cancer is not a feedback regulation of the upregulation of MMP, but is a vitally important and self-governed effect in colon cancer. All these results provided a new method to make prognosis prediction and suggested that TIMP1 could be a therapeutic marker.

## Conclusions

In summary, the present study identified that TIMP1 is overexpressed in colon tumor samples and that dysregulated TIMP1 expression, which occurs frequently in colon cancer, leads to tumor proliferation, metastasis and anti-apoptosis by FAK-PI3K/AKT and MAPK pathway. In animal experiments, the in vivo tumorigenicity and metastasis were weakened effectively by downregulation of TIMP1. These results suggest that TIMP1 signaling is a promising new molecular target for novel preventive and therapeutic strategies for this malignancy, and TIMP1 expression is critical for the progression and invasiveness of colon cancer. However, further explorations are needed to fully elucidate the molecular mechanisms governing TIMP1 gene dysregulation and its role in colon cancer progression.

## Additional files

Additional file 1: Figure S1. PPIs network from the STRING resource. TIMP1 was highly correlated with stars molecules, such as MMPs, EGFR, JUN, TGF-β and SMADs, etc. (TIF 3877 kb) Additional file 2: Figure S2. Inferring the functions of TIMP1 by Gene Set Enrichment Analysis (GSEA) in TCGA CRC dataset. Enrichment plots are shown for a set of activated genes of EMT, Apical junction, Apoptosis, IL2-STAT5, KRAS, Hypoxia, Angiogenesis, Hedgehog and TGF-βpathway. The enrichment score (ES, green line) represents the degree to which the gene set is over-represented at the top or bottom of the ranked list of genes. Black bars mean the position of genes belonging to the gene set in the ranked list of genes included in the analysis. (TIF 4894 kb) Additional file 3: Figure S3. Schematic representation for the TIMP1 regulatory pathway proposed in this study. TIMP1 activates FAK/PI3K-AKT and MAPK pathway, which facilitated AKT/ERK phosphorylation. This promotes the activation of BAD, cyclin D1, P21, P27 and other Transcription substrates, leading to potentiation of cell antiapoptosis, proliferation and invasion in CRC. (TIF 903 kb)
