# Supplementary figures and images for: TIMP1 is a prognostic marker for the progression and metastasis of colon cancer through FAK-PI3K/AKT and MAPK pathway
Source: J Exp Clin Cancer Res. 2016 Sep 20;35:148. doi: 10.1186/s13046-016-0427-7 (PMC5028967; doi:10.1186/s13046-016-0427-7)

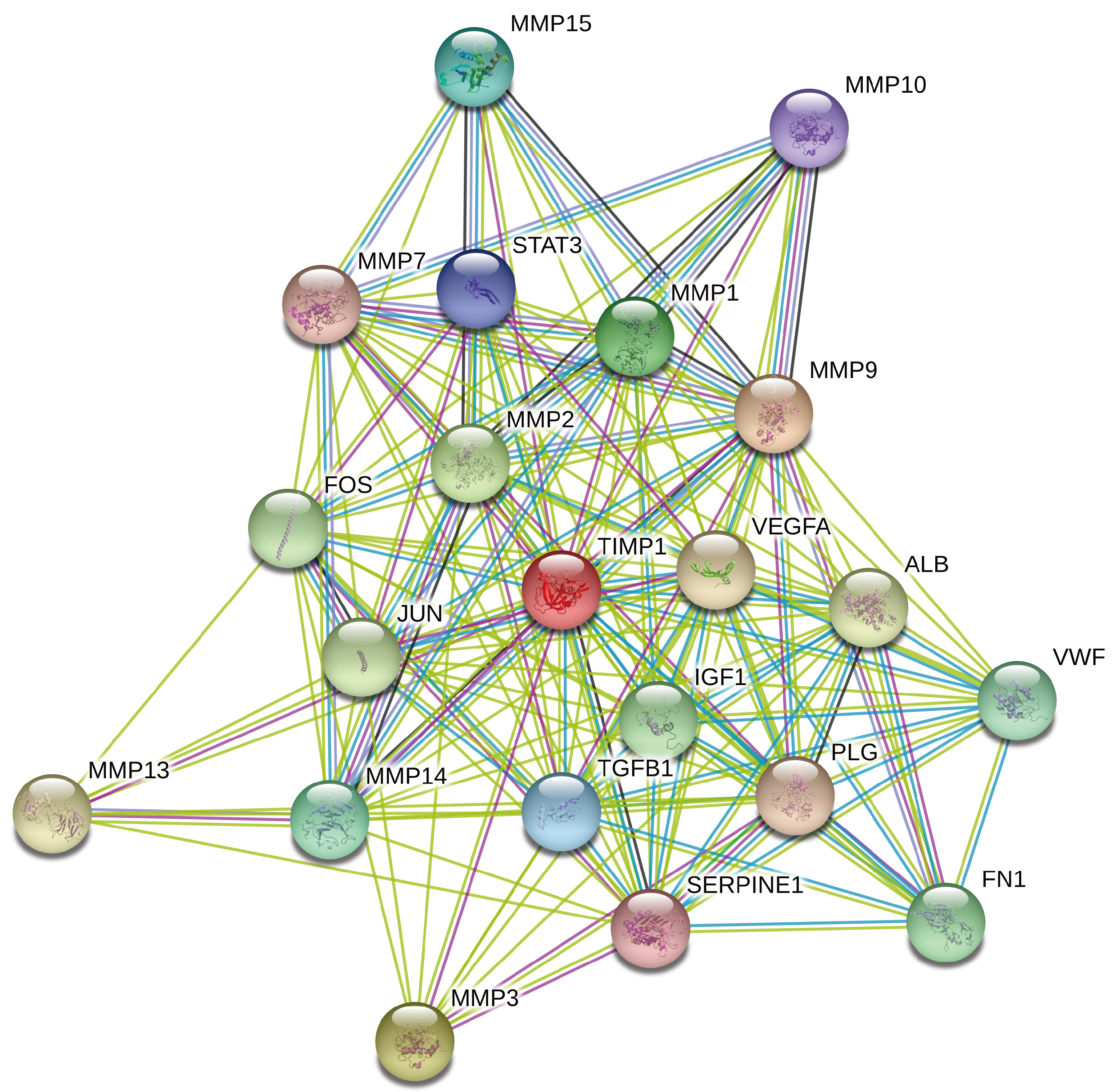

Supplement: Additional file 1: Figure S1. — PPIs network from the STRING resource. TIMP1 was highly correlated with stars molecules, such as MMPs, EGFR, JUN, TGF-β and SMADs, etc. (TIF 3877 kb) [file 13046_2016_427_MOESM1_ESM.tif]

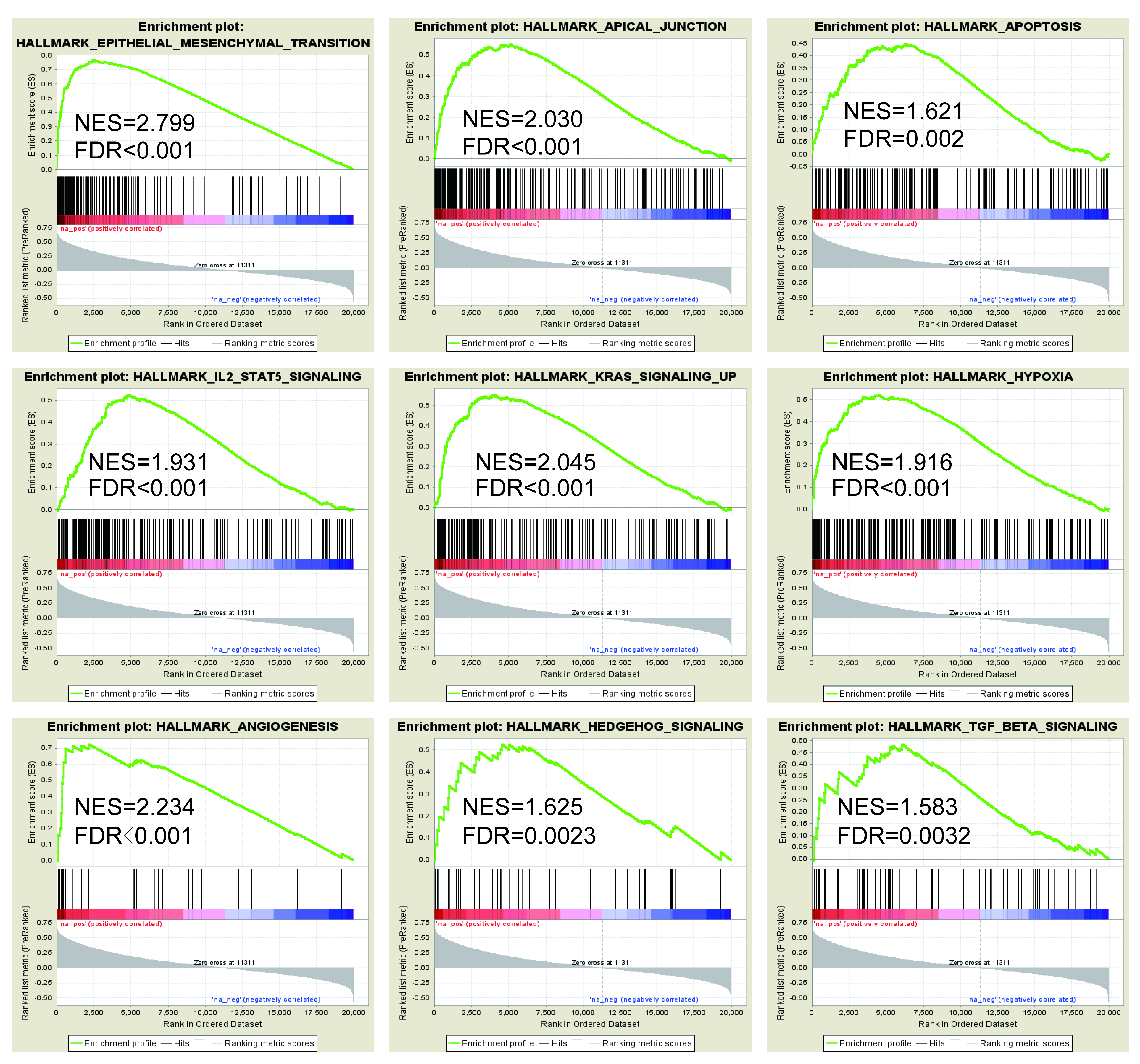

Supplement: Additional file 2: Figure S2. — Inferring the functions of TIMP1 by Gene Set Enrichment Analysis (GSEA) in TCGA CRC dataset. Enrichment plots are shown for a set of activated genes of EMT, Apical junction, Apoptosis, IL2-STAT5, KRAS, Hypoxia, Angiogenesis, Hedgehog and TGF-βpathway. The enrichment score (ES, green line) represents the degree to which the gene set is over-represented at the top or bottom of the ranked list of genes. Black bars mean the position of genes belonging to the gene set in the ranked list of genes included in the analysis. (TIF 4894 kb) [file 13046_2016_427_MOESM2_ESM.tif]

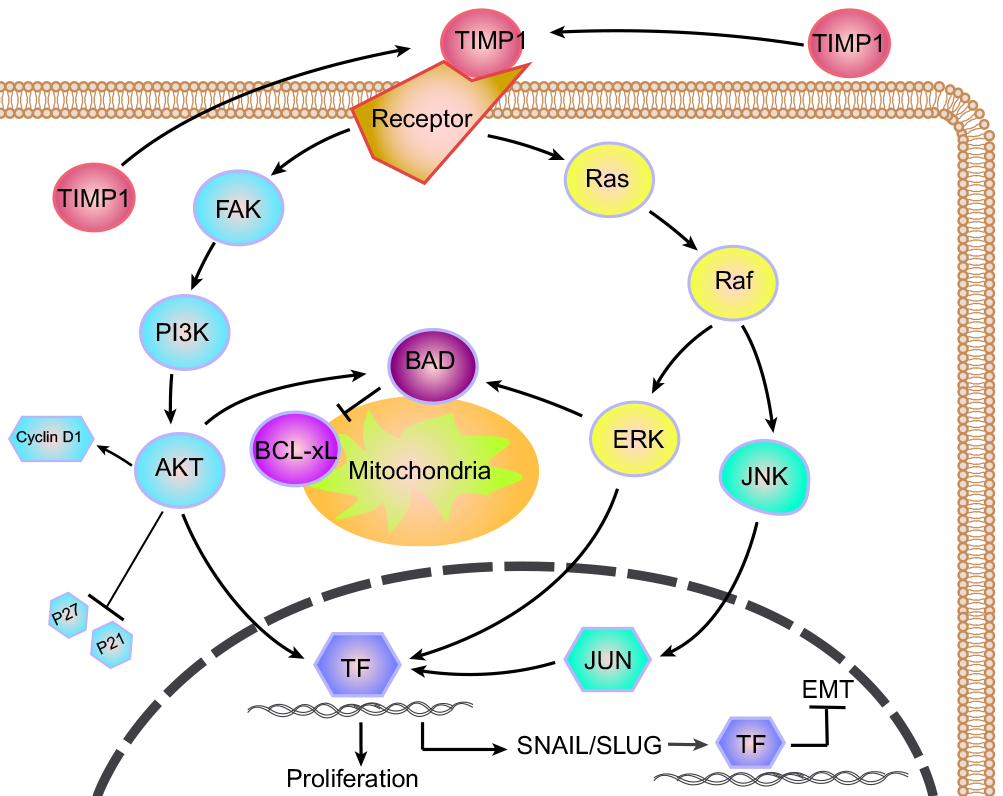

Supplement: Additional file 3: Figure S3. — Schematic representation for the TIMP1 regulatory pathway proposed in this study. TIMP1 activates FAK/PI3K-AKT and MAPK pathway, which facilitated AKT/ERK phosphorylation. This promotes the activation of BAD, cyclin D1, P21, P27 and other Transcription substrates, leading to potentiation of cell antiapoptosis, proliferation and invasion in CRC. (TIF 903 kb) [file 13046_2016_427_MOESM3_ESM.tif]
